# Corruption risks in COVID-19 vaccine deployment: lessons learned for future pandemic preparedness

**DOI:** 10.1186/s12992-025-01096-6

**Published:** 2025-03-07

**Authors:** Gul Saeed, Jillian Clare Kohler

**Affiliations:** 1 Leslie Dan Faculty of Pharmacy, University of Toronto, 144 College Street, Toronto, ON M5S 3M2 Canada; 2 Yale School of Public Health, Yale University, 60 College Street, New Haven, CT 06510 USA

**Keywords:** Accountability, Corruption, COVID-19, COVAX, Distribution, Vaccines, Procurement, Transparency

## Abstract

**Background:**

During the COVID-19 pandemic corruption risks were amplified in health systems globally, increasing health inequities within and between countries. During the pandemic, the deployment of COVID-19 vaccines, particularly concerning their procurement and distribution, had corruption risks given the large amounts of public and private funding allocated to them, the need for speed, the involvement of a high number of stakeholders, and often insufficient oversight. To explore this issue further, we conducted a descriptive, qualitative study of corruption risks in the COVID-19 vaccine deployment process.

**Methods:**

We conducted a descriptive, qualitative study triangulating two data sources between May and August 2022: (1) published academic and grey literature and (2) key informant interviews with representatives from organizations involved with the COVAX Facility, representatives from COVAX donor and recipient countries, and individuals with expert knowledge of the COVID-19 vaccine deployment process (e.g., consultants for international organizations involved in COVID-19 vaccine deployment, members of non-governmental organizations, etc.).

**Results:**

We identified 44 academic articles and policy documents and triangulated. Documentary data with 16 key informant interviews. A review of the literature identified several corruption risks in the international COVID-19 vaccine procurement and distribution process such as a lack of transparency in the vaccine procurement process; a lack of transparency in the operation of the COVAX Facility; a risk of bribery; and a risk of vaccine theft or the introduction of substandard and falsified vaccines at the point of distribution. Key informants further articulated concerns about a lack of transparency in vaccine pricing and contracts and the exclusion of civil society organizations from the vaccine deployment process. Reported anti-corruption, transparency, and accountability (ACTA) mechanisms implemented across the many levels of the vaccine procurement and distribution deployment included institutional oversight processes, blockchain-based supply-chain solutions, and civil society engagements.

**Conclusion:**

Public health emergencies require nimble and quick actions on the part of governments, international organizations and other actors Our study on the COVID-19 vaccine deployment process highlights the pressing need for more robust ACTA mechanisms to reduce corruption risks and ensure fair and equitable access to lifesaving vaccines for populations.

**Supplementary Information:**

The online version contains supplementary material available at 10.1186/s12992-025-01096-6.

## Background

Corruption is borderless; it is global in reach. Defined by Transparency International as “*the abuse of entrusted power for private gain*” [1] in the health system, specifically, corruption exacerbates inequities as it limits access to safe and effective health care services and products such as medicines and vaccines [2]. Corruption manifests in different forms amongst all types of institutional levels, sectors, and stakeholders [3, 4]. Given its technical and financial complexity, the pharmaceutical system is highly prone to corruption risks. The reasons why have been well researched and include the following: it is lucrative, has technical complexities and a number of complex decision points and involves multiple stakeholder groups in its governance, sometimes with competing interests [5]. For instance, the risk of corruption is greater in systems in which outside donors finance a significant portion of costs. Approximately 7% or 500 billion USD of global health expenditure is lost to corruption and fraud annually [6]. Corruption is an unequivocal threat to advancing the United Nations (UN) Sustainable Development Goal (SDG) #3 (Good Health and Wellbeing), as well as SDG 16 targets 16.5 and 16.6 (reducing corruption, bribery, and developing accountable and transparent institutions) [7, 8]. Above all, corruption results in inequitable access to health care services and products, leaving the most vulnerable populations at-risk for negative health outcomes.

Corruption showed up as a clear threat to public health goals during the COVID-19 pandemic [9, 10]. Indeed, there is ample research that illuminates the procurement process is highly susceptible to corruption during public health crises [11–13]. Specifically, the urgent need to procure large volumes of critical medical supplies and vaccines that are limited in supply may lead to bypassing regular rules and procedures and create opportunities for individual discretion, amplifying the risk of corruption [13, 14]. Corruption risks were amplified during the COVID-19 pandemic, as the rapid spread of the pandemic and the ensuing economic recession intensified competition for essential resources [13]. Publicly funded health products, such as vaccines, even in non-emergency situations, have corruption risks, particularly if sufficient anti-corruption, transparency and accountability (ACTA) mechanisms are not in place [15]. During the pandemic, there was a reported lack of transparency in COVID-19 vaccine contracts and clinical trial data, coupled with several countries’ pre-existing weak vaccine distribution systems, there were cases of nepotism and favouritism in vaccine access [4, 12, 16]. Ultimately, these factors exacerbate inequities in access to the COVID-19 vaccines, with the most disadvantaged populations disproportionately being impacted.

Research has consistently demonstrated that identifying corruption risks and implementing ACTA mechanisms are essential for reducing corruption risks in the pharmaceutical system [4, 11, 17]. While corruption risks in the procurement and distribution of health products have been studied in-depth, as described above [4, 15, 18], understanding the corruption risks that were present in the deployment of COVID-19 vaccines is vital to enhance future pandemic preparedness.

## The COVAX facility

The COVAX Facility, co-led by Gavi, the Vaccine Alliance (GAVI), the Coalition for Epidemic Preparedness Innovations (CEPI), and the World Health Organization (WHO), is one of the vaccine pillars of the Access to COVID-19 Tools (ACT) Accelerator that was established in 2020 to facilitate the global procurement of COVID-19 vaccines and help advance equitable access to these lifesaving vaccines for all populations [19, 20].

Running from February 2021 until December 2023, COVAX was the first initiative of its kind on a global scale. COVAX was a public-private partnership (PPP) model that included the engagement of global health entities, regulatory bodies, pharmaceutical firms, and private philanthropic organizations [21]. The COVAX Facility had a complex governance structure to promote accountability, transparency, and diverse representation. Alongside international organizations co-leading the initiative, UNICEF served as a key delivery partner for COVAX, while in the Americas region, the PAHO Revolving Fund was designated as the official procurement agent for COVAX [22]. In terms of Facility Management, the Office of the COVAX Facility, overseen by Gavi, managed operations such as vaccine procurement and country engagement [23]. Facility governance was overseen by the Gavi Board and supported by the Audit and Finance Committee (AFC) for financial oversight and the Market-Sensitive Decisions Committee (MSDC) for business term approvals with vaccine manufacturers [23]. The COVAX Shareholders Council guided operations for self-financing economies [23]. Technical and advisory bodies like the Independent Product Group (IPG) and the Procurement Reference Group (PRG) were tasked with the responsibility of ensuring balanced vaccine inclusion and portfolio management [23]. The Joint Allocation Taskforce (JAT) supported by the Independent Allocation of Vaccines Group (IAVG) prepared allocation proposals based on data. Operational coordination occurred through the COVAX Coordination Meeting (CCM), aligning efforts across COVAX’s stakeholders [23].

COVAX additionally assured participating countries access to a diverse range of vaccine candidates targeting the SARS-COV-2 virus. Countries also retained the option to procure vaccines independently from sources outside the COVAX Facility. Despite its ambitious goals and good intentions, COVAX was a monumental global effort that sought to overcome unprecedented challenges in vaccine distribution. However, by late 2021, it became evident that the COVAX Facility was not functioning as anticipated, failing to achieve its target of distributing 2 billion doses by the end of 2021 [24]. There are many reasons why this failure happened, but in our research, we sought to investigate the corruption risks that were present in the vaccine deployment processes and to identify critical lessons for future pandemic preparedness.

### Study rationale

The COVID-19 pandemic underscored the critical importance of transparency and accountability in global health initiatives, particularly in vaccine deployment. A growing body of literature has shed light on the corruption risks in the deployment of COVID-19 vaccines and the role of ACTA mechanisms in reducing these risks [25]. Using the unprecedented international efforts in COVID-19 vaccine procurement and distribution as a case study, our research aimed to uncover vulnerabilities and corruption risks that compromised equitable vaccine access. By identifying these challenges, we sought to provide actionable insights to strengthen preparedness for future pandemics. Specifically, we (1) identified the risks of corruption in the procurement and distribution of COVID-19 vaccines at international and national levels and (2) examined the ACTA mechanisms implemented in the COVID-19 vaccine procurement and distribution processes.

## Methods

We conducted a descriptive, qualitative study triangulating two data sources:

(1) published academic and grey literature and (2) key informant interviews with representatives from organizations involved with the COVAX Facility, representatives from COVAX donor and recipient countries (India, Kenya, Nigeria), and individuals with expert knowledge of the COVID-19 vaccine deployment process (e.g., consultants for international organizations involved in COVID-19 vaccine deployment, members of NGOs, etc.). This study was reviewed and approved by the University of Toronto’s Research Ethics Board (Protocol Reference #00042039); all methods were performed in accordance with relevant guidelines and regulations. Ethics Approval was obtained on February 4, 2022, in advance of participant recruitment, which began in May 2022. Before each interview, participants provided written informed consent.

## Targeted literature review and data extraction

Documents with a focus on corruption and COVID-19 vaccine procurement and distribution were identified from academic databases and grey literature sources. Academic literature was extracted from the following electronic databases: OVID Medline, Scopus, Web of Science, and Proquest PAIS Index. Our search strategy was developed using different combinations of three key concepts: “Corruption”, “COVID-19 Vaccines” and “Procurement and Distribution” in Medline. A modified OVID expert search was used for the “COVID-19 vaccines” concept [26]. For “Corruption” and “Procurement and Distribution,” subject headings and text word searches of synonyms reported in the literature were used along with truncations and adjacencies [4, 12]. The search was then adapted for other databases.

Grey literature was extracted from targeted website searches of international organizations (i.e., CEPI, GAVI, UNICEF, UNODC, WHO, and the World Bank Group [hereafter the World Bank]) and non-governmental organizations (NGOs) (i.e., Transparency International, U4 anti-corruption resource centre) websites. Target searches were conducted utilizing search terms related to corruption, COVID-19 vaccines, and procurement and distribution. We also ran our search strategies on the Google Scholar search engine to capture relevant peer-reviewed and grey literature not captured during the searches. All literature searches were initially conducted in June 2022 and updated in April 2024. Additional articles that were already known to the author or were recommended by key informants were also included.

**Fig. 1:**
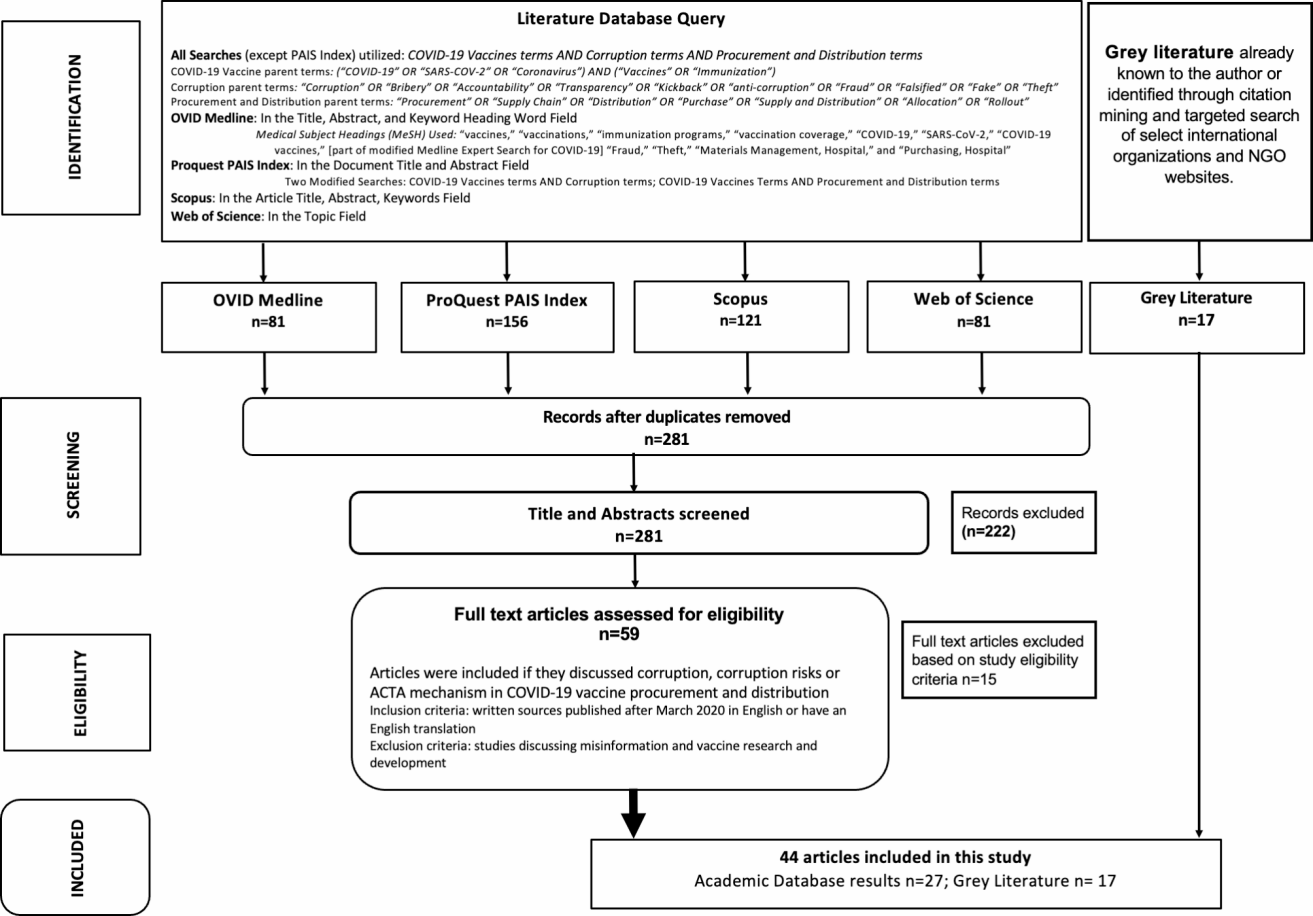
Flow chart of literature review methodology

All identified documents were retrieved from each database and uploaded to Covidence for automatic deduplication. Any remaining duplicates were removed manually. A first round of eligibility screening was conducted on title and abstracts against the inclusion/exclusion criteria (see Fig. 1). Our inclusion criteria included: (i) articles that focused explicitly on corruption or ACTA mechanisms in the context of COVID-19 vaccine procurement and distribution and (ii) articles published between March 2020 to December 2022, reflecting the years of the COVID-19 pandemic. March 2020 was selected as the starting point because that is when COVID-19 was declared a pandemic by the WHO. Exclusion criteria included: (i) sources discussing misinformation, vaccine hesitancy, and COVID-19 vaccine research and development, (ii) non-English sources, (iii) non-written sources, such as podcasts or videos. If abstracts met the inclusion criteria, they underwent a full-text review prior to the extraction of descriptive data by two research team members.

Descriptive data extracted from each article included the following: title, author, source, year of publication, type of publication, objectives, methodology, geographic location, reported case(s) of corruption, reported corruption risks, level of corruption risk (international, national, regional, or local), reported ACTA mechanisms, and vaccine candidates involved.

## Key informant recruitment and interviews

We conducted 16 semi-structured key informant interviews via Zoom between May 10, 2022 to August 4, 2022. Guided by purposive and snowball sampling strategies [27], we selected key informants based on their involvement in the COVID-19 vaccine deployment process. Informants included representatives from international organizations, representatives from COVAX donor and recipient countries, and members of NGOs. Before each interview, key informants provided their written consent. Interviews were approximately 30 and 45 min in duration and were conducted in English (see Appendix A for interview guide). Detailed notes were taken by both the interviewer and another member of the research team to capture key details and direct quotes when possible. After each interview, the notes were emailed to each key informant for member checking to ensure that they accurately captured what the individual had shared during the interview. Subsequently, notes were de-identified by the interviewer.

Interview notes were coded using the qualitative analysis software NVivo 12 and then verified by a second coder. We employed a combination of inductive and deductive thematic analysis [28] to identify common themes across the dataset. Our initial coding framework was created using deductive thematic analysis, where our research objectives and targeted literature review were used to the codes [28, 29]. We used inductive thematic analysis, a data-driven approach, to add nuance and specificity to the coding framework by focusing on emerging insights from key informants [29]. Throughout the data analysis process, the coding framework was modified as new codes were added when data did not fit into existing codes. Subsequently, the coded data were used to develop theme memos, with similar codes being organized and grouped into potential themes and subthemes.

## Results

We identified 27 articles and 17 grey literature documents describing corruption or ACTA mechanisms in the international COVID-19 vaccine procurement and distribution processes for final review (see Fig. 1). Of the 50 individuals invited to participate in interviews, 22 responded and 16 consented to participate (see Table 1). Several noted that their employing organizations had non-disclosure agreements in place related to the deployment of COVID-19 vaccines and thus could not participate or could only share limited information.

Most of the key informants were employees at international organizations directly involved in the global deployment of COVID-19 vaccines. The represented international organizations included GAVI and the WHO, who co-led the COVAX initiative, with GAVI also being involved in the monitoring of the COVID-19 vaccine deployment process at the country-level; UNICEF, who on behalf of COVAX was involved in the delivery of COVID-19 vaccines to countries and updated the COVID-19 Market Dashboard, a public dashboard comprising information about how allocation was operationalized and manufacturing agreements and reported vaccine prices; the UNDP that provided technical assistance to countries to strengthen their vaccine delivery and health systems vis-à-vis the implementation of digital solutions; and the World Bank that financed COVID-19 medical equipment and vaccines.

At the country-level, some of the key informants were employees at GAVI and UNDP country offices or with civil society organizations in COVAX recipient countries, namely India, Kenya, and Nigeria. A few key informants were from NGOs based in high-income countries that were involved in monitoring the deployment of COVID-19 vaccines in low- and middle- income countries (LMICs).

**Table 1:** Key informant characteristics

Key Informant Type	Number of Key Informants	Number of Individuals Invited to Participate
Representatives from international organizations: GAVI, UNICEF, WHO and the World Bank	9	20
Representatives from COVAX recipient countries: India, Kenya, and Nigeria	India (2), Kenya (1), And Nigeria (2)	15
NGO employee: Accountability Research Center and Partnership for Transparency Fund	2	15
**Total number of key informants**	16	50

In the following section, we present lessons learned from the international procurement and distribution of COVID-19 vaccines based on findings from the rapid literature review and key informant interviews. These insights are organized into four thematic areas: (1) corruption risks in the COVID-19 vaccine procurement and distribution processes; (2) corruption cases during COVID-19; (3) impact of corruption on the deployment of COVID-19 vaccines; and (4) ACTA mechanisms in the deployment of COVID-19 vaccines. **The quotes provided by the key informants in this section are verbatim.**

### Corruption risks in the COVID-19 vaccine procurement and distribution processes

#### Vaccine procurement

Prior to the launch of the global deployment of COVID-19 vaccines, the UNODC (2020) identified several corruption risks at each stage of the vaccine procurement process (pre-bidding, bidding, and post-bidding) (see Table 2) [12]. In the pre-bidding stage, corruption risks can include tailoring tender criteria to meet the preferences of specific bidders and creating a falsified needs assessment of public vaccine demand [12]. The UNODC (2020) further emphasized that one of the key risks in vaccine deployment was that government officials may be open to receiving bribes during the initial needs assessment to benefit certain companies by pre-determining which vaccines are purchased, how many, and from whom [12]. During the bidding stage, the UNODC identified that there may be the risk of manufacturers/suppliers-origin bribes, as well as collusion between bidders in the vaccine market to inflate prices [12]. Likewise, Rose-Ackerman (2021) described that in emergencies, time constraints and a shortage of qualified suppliers can compromise the transparency and clarity of the procurement contracting process, fostering conditions conducive to bid-rigging, price-gouging, and fraudulent activities by firms [13]. In contrast to the corruption risks identified prior to the launch of the COVID-19 vaccines, we found that most countries did not go through the bidding stage as they purchased COVID-19 vaccines directly from pharmaceutical companies, through regional entities like the European Union, or received them through COVAX [30]. Consequently, this likely minimized the procurement process’ vulnerability to the corruption risks typically present in the procurement process. Nevertheless, skipping the competitive procurement process, which aims to solicit fair, impartial, and competitive bids, due to the urgent need to acquire vaccines can create room for corruption. One key informant commented from an NGO:

*One of the major causes of corruption in this whole deployment process are suppliers. Corruption is mainly at procurement rather than service/delivery*.

While we assume certain corruption risks, such as those related to the bidding process, are reduced by turning to an international organization for the purchase of vaccines, it does not entirely eliminate other potential areas of corruption in the procurement process. For instance, overall transparency in vaccine pricing negotiations between manufacturers and countries was found to be lacking, with a key informant working with the COVAX initiative commenting: “*Of course*,* the lack of transparency regarding price of vaccines at the beginning was a huge window for corruption.*” Indeed, a lack of transparency in vaccine price negotiations can lead to corruption risks in pricing as all relevant information is not made publicly accessible which reduces the probability of detecting corrupt acts, including inflated vaccine contracts.

A key informant who consulted for an international organization further explained that it was difficult to make informed purchasing decisions due to the lack of publicly available information:

All decisions are very opaque. We don’t even have the prices. We need to know how decisions were made and what were the other available option.…so it’s difficult to evaluate at times of crises, we don’t have coordinates to see how these decisions were made. I don’t know if a country paid $10 or $2 because nothing to compare it against….

Another key informant who previously worked with an international organization noted that the risk of corruption also increased in the procurement of COVID-19 vaccines in different parts of the world because:

Many intermediate agencies popped up…[and governments were] directly purchasing vaccines from them.…That is not direct purchase through manufacturer, and this increases corruption risk. If the deal is directly from manufacturer, you have some security of quality and price.

In the post-bidding stage, perceived corruption risks identified by the UNODC (2020) included falsified invoicing and a lack of due diligence when introducing last-minute amendments to final vaccine procurement contracts, as well as non-delivery or under-delivery of vaccines [12, 13]. Since the launch of the global deployment of COVID-19 vaccines, there have been reports that vaccine contracts between governments and manufacturers have given manufacturers “leeway on delivery dates and pricing,” with promises that most information in these contracts will not be made public [30]. Due to the lack of transparency of these vaccine deals, the exact nature of corruption remains unclear.

A lack of public disclosure in the final contracts negotiated between governments and vaccine manufacturers was also identified as key source of corruption risk, either as a mechanism to shield individual transactions from public scrutiny [31] or to facilitate pricing opacity between different purchasing countries [32, 33]. In reference to contracts executed by a country in Latin America, one key informant who works at an NGO commented: “*…the government was even more opaque in the procurement of COVID vaccines than they were for other drugs like for HIV and diabetes*, etc.”

Another key informant from an NGO shared that in Nigeria:

…the government and the NCDC [Nigeria Centre for Disease Control] were not putting out data on what was procured vs. donated. Vaccines were donated and government is also saying that cash was used for procurement of vaccines and other medical facilities, but they do not have data to show what funds went to the purchasing of vaccines, what went to buying other medical equipment. Those details are not in public domain for now. No definite data on how many were purchased or donated.

## Vaccine distribution

Corruption can manifest in many different forms during the vaccine distribution process. As reported in GAVI’s Risk & Assurance Report from 2020 to 2021, there was theft and diversion of COVID-19 vaccines during the pandemic in many countries [34, 35]. Theft and diversion can occur during transportation, or after vaccines have reached distribution sites such as public health facilities [12, 31]. Diversion occurs when vaccines are re-routed to non-target groups within a country or sold through exploitative transactions in the black market [12, 34, 35]. Manufacturing, transport, and health facilities staff may steal vaccines from inventory for resale [12, 31], while consumers may try to bribe bureaucrats to acquire additional doses for resale on grey markets [36, 37].

For example, in Uganda, it was reported that AstraZeneca vaccines were stolen from healthcare facilities by healthcare workers and diverted to the private market for resale [38]. Similar cases of theft were reported in the United States [39, 40]. Additionally, health professionals asked for or were offered bribes from patients to access COVID-19 vaccines before their official eligibility [41]. This allows room for favoritism and nepotism, benefiting those who are wealthy or have the right connections whilst leaving the poor and vulnerable groups without access to these lifesaving vaccines [12, 40]. For instance, a doctor in Beverly Hills reported that he was offered more than USD 10,000 from persons, including those from the entertainment industry, who wanted to get vaccinated [41]. Moreover, multiple cases were reported in Uganda and Bangladesh, where patients were asked to pay healthcare workers or facilities for vaccines that were supposed to be free of charge, some of which included falsified or substandard vaccines. A key informant working at an NGO conducting local vaccine distribution monitoring projects also shared:

VIPs got vaccines first when it was supposed to be the most vulnerable populations, aged and hard-to-reach populations first, but people with access got VIP treatment. Serious inequities within and among countries as well.

This was echoed by an independent consultant for an international organization, who remarked that:

*“How allocation is done based on criteria defined by government is not always*

*followed by countries.…Initially*,* there were stories of falsification and supply coming from many different places and some workers taking away vaccines for themselves. Slowly as vaccine coverage improved*,* this calmed down.”*

Finally, an employee at an international organization shared:

…the parliament unit got vaccines before everyone else instead of using the [official] vaccine platform.

What is more, in Bangladesh, criminal networks exploited the vaccination registration system and accepted bribes from vaccine seekers to allow access to earlier vaccination dates [38]. Organized criminal groups may also take advantage of corruption vulnerabilities in the vaccine deployment process to traffic substandard vaccines or manufacture falsified products [12]. Falsified COVID-19 vaccines have been identified in Uganda, India, Myanmar, Mexico, the Philippines, and Nigeria [38, 40]. In Zambia, criminals falsely claimed to be health workers, accepted bribes from patients, and subsequently, provided them with falsified vaccines [38].

**Table 2:** Overview of corruption risks in the COVID-19 vaccine procurement and distribution processes

	Identified corruption risks	Key points	Source of information
**Vaccine procurement**			
Pre-bidding stage	- Tailoring tender criteria to specific bidders. - Falsified needs assessments of vaccine demand. - Bribery to influence decisions on vaccine type, quantity, and source.	- Needs assessments could be manipulated to benefit specific companies. - Officials may pre-determine vaccine procurement for personal or political gain.	Literature review: - United Nations Office on Drugs and Crime (2021)
Bidding stage	- Manufacturer/supplier-origin bribes. - Collusion among bidders to inflate prices. - Lack of transparency in the contracting process due to time constraints.	- Bid-rigging, price-gouging, and fraudulent activities thrive in emergencies. - Most countries skipped competitive bidding, reducing some risks but introducing others like limited oversight and opaque deals.	Key informant interviews and Literature review: - Apuzzo & Gebrekidan (2021) - Rose -Ackerman (2021) - United Nations Office on Drugs and Crime (2021)
Post-bidding stage	- Lack of due diligence in last-minute amendments to contracts. - Falsified invoicing. - Non-delivery or under-delivery of vaccines. - Vaccine contracts often lacked public disclosure, shielding transactions from scrutiny.	- Contracts offered leeway on delivery timelines, pricing, and other critical terms. - Governments lacked transparency in differentiating procured vaccines from donated ones.	Key informant interviews and Literature review: - Apuzzo & Gebrekidan (2021) - Hussman et al. (2021) - Rose-Ackerman (2021) - Sung et al. (2021) - United Nations Office on Drugs and Crime (2021) - Usman et al. (2022)
**Vaccine distribution**			
	- Misallocation or diversion of vaccines. - Lack of tracking systems to monitor vaccine distribution. - Nepotism or favoritism in the allocation of vaccines to certain groups.	- Some countries did not disclose whether vaccines were procured or donated, creating accountability issues. - Unequal vaccine allocation due to governance and logistical failures.	Key informant interviews and Literature review: - Gavi, the Vaccine Alliance (2020) - Gavi, the Vaccine Alliance (2021) - Goel et al. (2021) - Farzanegan and Hofmann (2021) - Hauck (2021) - Rahman (2021) - Transparency International (2022) - United Nations Office on Drugs and Crime (2021) - Usman et al. (2022)

### Deployment of COVID-19 vaccines through the COVAX Facility

The COVAX Facility played a crucial role in global vaccine deployment but when distributing vaccines to countries had corruption risks due to governance gaps. Its experience provides some key lessons learned related to corruption risks and vaccine deployment in a global health emergency. Between February 2021 to December 2023, COVAX shipped approximately 1.99 billion doses of the COVID-19 vaccines to 146 countries, with the United States, Germany, and France being a few of its biggest donors based on publicly available data [41]. COVAX adopted a hybrid public-private partnership model to facilitate vaccine distribution [42]. While this structure allowed for resource pooling and rapid response, some stakeholders noted that it also created opportunities for wealthier nations to dominate the vaccine donation space for political and economic gains [43]. When coupled with the COVAX Facility’s lack of enforcement ability against governments that fail to abide by its dose-sharing principles, the risk of political exploitation that undermines the operational objectives of the organization severely increases. A key informant from an international organization explained that at COVAX:

Nothing particular was in place regarding accountability except what was in agreements.…It was a matter of power allocation. It is crucial to determine who has power and to what extent. The limits of the power were not really hashed out with the COVAX initiative. COVAX was too naïve to think everyone will collaborate and work together to end pandemic, but nationalism, profits, and lack of public health interest came into play. COVAX overestimated humanity of institutions, governments, and the private sector.

The absence of robust oversight mechanisms at the national level was a critical challenge. While COVAX facilitated vaccine procurement and allocation, informants noted gaps in ensuring transparency and accountability in vaccine distribution within recipient countries. A key informant working with COVAX shared: “*…we don’t know what happens with the vaccines at the national-level and we are unsure how the governments are distributing the vaccines*.” Further noting that “*COVAX could have important role at country-level and play critical role in local distribution of vaccines.*” Due to COVAX’s lack of oversight at the country-level, it created opportunities for corruption, especially in countries that had weak governance structures in place.

Internally, COVAX demonstrated transparency, providing tools to share information on vaccine acquisitions and distribution. However, access to this information was not always available to persons in recipient countries. A COVAX staff member noted:

There were a lot of tools that enhanced transparency within the organization, but it was a question of who was benefitting from the transparency of information. For example, information was available to everyone across the organization regarding vaccine acquisition and distribution, but this information was not available to countries due to too many politics behind this.

Likewise, in their qualitative study, Gorodensky et al. (2024) found that while COVAX established policies to promote transparency in vaccine pricing, delivery schedules, and clinical trial data sharing, these measures were not consistently enforced, rendering them largely ineffective [43]. As an example, COVAX provided some transparency regarding allocation decisions, but the algorithm driving those decisions remained undisclosed, hindering full transparency [43].

COVAX’s experience illustrates the inherent challenges of managing a large-scale, multi-stakeholder initiative in a rapidly evolving global health crisis. These challenges highlight the importance of balancing speed and equity with transparency and accountability mechanisms.

### Corruption cases during COVID-19

During the interviews, some key informants expressed that most cases of corruption in the COVID-19 vaccine deployment process “*so far appear to have been sporadic and opportunist*,” occurring at the individual level rather than the systemic or institutional level. This was supported by a Transparency International (2022) study, which identified cases of bribery, embezzlement, and fraud in Bangladesh, Uganda, and Zambia during COVID-19 [38]. Further, in Brazil, the head of the health ministry import division reported pressure to accept a $315 million deal with India’s Bharat Biotech for 20 million COVID-19 vaccine doses. Then President Bolsonaro was alerted to irregularities in March, including early and expensive procurement of Covaxin despite less advanced clinical trials [44]. Rahman (2021) also reported nepotism and favoritism in vaccine distribution in Argentina, Brazil, Canada, Kenya, Peru, Poland, and Spain, where political leaders and wealthy individuals have been seen cutting the line [40]. For example, one key informant from an NGO reported:

Government stored a lot of covid materials in warehouses instead of sharing with citizens and they were waiting to give them to their party loyalists or saving it to use it during campaigns to get votes.… [There was a] case where certain public official used it as a souvenir. She had her picture on the supplies and gave it to loyalists.

Other reported cases of corruption included individuals impersonating frontline health workers to get access to vaccines, theft of vaccines, embezzlement, vaccine hoarding, and misappropriation of goods and funds in several countries including, but not limited to the Bangladesh, Colombia, Malawi, South Africa, the United Kingdom, the United States [40, 45]. Criminal networks have also reportedly stolen expired vaccines from authentic sources with the help of corrupt healthcare personnel and officials to sell online [40]. A key informant who works at an international organization country office shared that there were:

…reports of falsified vaccines in one of cities and the warehouse was raided. There had been a worker from inside a manufacturing facility that had provided templates of vials so mini production line was created to produce fake vaccines.…This one particular case was quite elaborate in terms of labelling vaccines and there were clear distinctions between what actual vial looked like vs. fake, but a lay person would not have known. The actions of authorities were highly publicised to let population know to not buy anything from black market.

At the institutional level, cases of widespread procurement mismanagement and corruption among public officials and departments have resulted in reduced levels of public trust in government institutions [46]. A key informant from Nigeria reported that:

…mistrust in government plays a huge role because Nigerians think COVID is a ploy by government to get funds for their personal use and don’t believe anything that is procured. Broken trust contributed largely to why people did not respond to or accept the vaccine and all the processes involved in this process. Mistrust happened because of other instances… The Managerial Ministry that was established to respond to the emergency situation came out at some point and said that they spent a very huge amount of money on feeding school children but at time of COVID no child was in school, children were at home. Basically, they were saying we spent over 2 million in feeding children who were at home. It didn’t add up. This indicated big mismanagement and fraud.

These corruption cases during the COVID-19 vaccine deployment process, reported across various countries and regions, highlight the diverse scales and forms of corruption that can arise during a global health crisis, underscoring the critical need for transparent, efficient, and accountable systems in future pandemic responses.

### Impact of corruption on the deployment of COVID-19 vaccines

Corruption in the COVID-19 deployment processes resulted in inequitable distribution of these life-saving vaccines, leaving populations in LMICs vulnerable to emerging variants of the virus [33]. High-income countries hoarded excess vaccines beyond their population’s needs, creating stark disparities in vaccine distribution during a time of limited global supply and heightened demand, especially in the early stages of the rollout [32]. With low supply and high global demand particularly during early stages of the vaccine rollout [37], this mismatch has been called a “catastrophic moral failure” and an “infringement upon human rights” [33]. The resulting inequitable distribution exacerbated pre-existing health, social, and economic inequities in LMICs [47, 48].

In some cases, vaccine supply was not a primary concern; instead, vaccine hesitancy, low public trust in government, and a lack of motivation to get vaccinated due to the low cost-effectiveness of procuring vaccines in certain countries hindered vaccine demand. More specifically, corruption in the public coordination of vaccine procurement and deployment reduced trust in government authorities, further fueling vaccine hesitancy [49]. As one key informant from an NGO noted, “*so many people don’t trust the government*.” Conversely, populations with higher levels of public trust—bolstered by lower levels of public sector corruption, transparency, and effective communication—reported higher vaccine acceptance and uptake [50, 51]. For example, a key informant based in Kenya explained that while Kenya had a supply of COVID-19 vaccines, the demand for it was very low compared to routine vaccines:

…where there is lack of trust in government, there are demand challenges (i.e., low demand for COVID vaccines due to government mistrust and vice-versa). For example, in Kenya, COVID-19 vaccination coverage is 25–26% out of around 50 million people, but for routine vaccinations it is 80%.

In countries with lower rates of compliance, low demand for the COVID-19 vaccines, and an inability to get the vaccines to the right place before their expiration date, most vaccines went to waste. A key informant from Nigeria shared that in their country: *“…people did not demand for it; over 90% of both donated and procured [vaccines] were destroyed…”*.

Relatedly, key informants noted that a lack of public trust resulting from corruption extended beyond the national level, with corruption risks undermining both the public’s and government’s trust in global initiatives like COVAX. A key informant from COVAX explained:

*“Risk of corruption leads to public’s lack of trust in these kinds of global initiatives. COVAX is largely an NGO trying to participate to make COVID vaccines accessible and affordable to countries. This was a big opportunity to tell the world that NGOs can run this kind of equitable initiative. However*,* COVAX couldn’t do it in the end and the initiative failed. COVAX had a financial crisis at the end*,* primarily because donations were keeping it alive. This type of result gets lack of trust from governments and end-users.”*

Importantly, all key informants identified local populations, particularly those who are the most vulnerable, as the key stakeholder group that is most adversely affected by corruption within the COVID-19 procurement and distribution chain.

*“If a country is only getting vaccines that are expiring in a month*,* the most vulnerable people there are unlikely to get them so it’s just whatever the barriers are they are going to be worse for people who are worse off.”*–Key informant from an NGO.

To the extent that corruption means that people do not get their vaccines, ultimately disadvantaged person is the person who doesn’t get the vaccine they are entitled to.

–Key informant from an NGO.

*“I would say beneficiaries and taxpayers are most affected. Beneficiaries*,* they won’t get their fair share at same time and the right place. Those same people will be taxpayers so there is a double jeopardy or double negative for beneficiaries.”*–Key informant working at country office in India.

### ACTA mechanisms in the deployment of COVID-19 vaccines: Lessons learned and recommendations for pandemic preparedness

ACTA mechanisms need to be embedded in health and pharmaceutical systems prior to any public health emergency to reduce corruption risks. The integration of ACTA mechanisms not only reduces corruption risks but also has significant spillover benefits, such as improving transparency, enhancing public trust, promoting efficient resource allocation, and ensuring better access to essential health services and medications for populations. For example, in the Philippines, transparency was a key to boosting public confidence in COVID-19 vaccine efficacy and safety [52]. Hinson et al. (2021) also highlighted the need for transparency in vaccine negotiations and decisions in Ghana to build trust in the government, promote vaccine acceptance, and support effective vaccine delivery [53]. While transparency is critical to enable corruption to be detected, accountability is an important follow up as it holds corrupt actors responsible for their behavior. Indeed, a key informant from an NGO explained that:

Transparency isn’t everything, it is a first step. Transparency has to lead to something actionable and there needs to be ways of addressing it. There has to be an openness to addressing it which could be shown by releasing the information, willing to make corrections along the way, and do things differently.

Despite this, we find that implemented and proposed ACTA mechanisms involved in the international COVID-19 procurement and distribution system primarily centered around enhancing institutional and procedural transparency, rather than heightening accountability standards. Nearly all sources focused on transparency mechanisms in the international COVID-19 procurement and distribution system. However, some key informants noted that GAVI monitoring agents had been placed in approximately 26 hard-to-reach countries to specifically evaluate potential equity and accountability issues. However, one key informant shared that monitoring agents were placed “*halfway through the pandemic. They were too little too late*” and “*should have been placed at the inception of the vaccine distribution phase.*” At the domestic level, another key informant from an international organization noted that:

[Healthcare] workers have to account for shots they have given, who they have given and how much wastage there is. This basic accountability principle was mandated in most LMICs.

Despite the importance of accountability mechanisms illuminated by key informants, several explained that “*things are getting worse in terms of accountability*” during the pandemic, attributing this trend to governments and prominent organizations reducing or halting oversight activities. This was especially prominent at the national level because of “*not having enough capacity*,* not having enough staff*.”

A key informant working at COVAX further described that the lack of transparency from governments about what they were receiving from donors “hindered accountability at the national level because citizens did not know” what was coming into their country and how it was being used. Similarly, the literature highlighted that oversight mechanisms and anti-corruption infrastructure, which are typically used to identify and eliminate intentionally ineffective, substandard, or hazardous medical products from supply chains—such as detection by national regulatory authorities (NRAs) and rigorous procurement procedures—may be deprioritized or overlooked in efforts to expedite countries’ responses or due to the overwhelming global demand [25, 54]. As another example, during the COVID-19 pandemic, starting in March 2020, the US Food and Drug Administration (FDA) reduced accountability measures by limiting domestic and foreign inspections to ensure the safety of its employees. The FDA adopted alternative inspection methods, such as reviewing reports from foreign regulators, to maintain some oversight of drug manufacturing quality while inspections were on hold. However, these methods are not always equivalent to a full FDA inspection [55].

### Oversight committees

The establishment of oversight committees, specifically designed to monitor and manage vaccine programs emerged as a critical lesson from the COVID-19 pandemic. For future public health emergencies, creating specialized committees to oversee the allocation of funds, procurement of vaccines, and their equitable distribution is essential [12]. Such committees, particularly at the provincial or district level, can foster public trust among local communities and ensure fair and transparent vaccine distribution [46]. For these committees to remain effective and accountable, regular audits must be conducted, with findings made publicly accessible. This approach not only fosters greater accountability but also strengthens public confidence in the integrity of pandemic preparedness and response systems.

### Financial auditing

The allocation of substantial funding for COVID-19 vaccines underscores the importance of rigorous financial auditing processes to ensure transparency and accountability in vaccine financing and procurement [12]. Robust auditing frameworks are also needed to track the flow and use of funds effectively. For example, the Philippines established a Joint Congressional Oversight Committee to review weekly reports to Congress about the allocation and use of COVID-19 funds [12]. Similarly, in Thailand, the National Health Security Office was subjected to internal audits on its procurement activities during COVID-19, enabling disbursements made to vaccine manufacturers to be publicly tracked through its Ministry of Public Health immunization information centre [56].

Key informants emphasized the critical role of regular audits in fostering accountability. One NGO representative underscored the importance of an annual audit in their country, sharing that: “*having that information out and possibly use that as a way for people to be held accountable.*” Another key informant, who serves as a consultant for an international organization, stressed the need for auditing measures within countries, noting that each international organization and multilateral organization has its internal structures comprising “*inspector general and internal audit and governing board.…this stops once we are in the country because then there are the country’s governing structures*…” For future pandemic preparedness, implementing and publicizing financial audits at all levels is essential to strengthen trust, prevent misuse of funds, and ensure equitable vaccine access.

### Procurement contract transparency

Many key informants advocated for greater transparency in vaccine supply contracts brokered between manufacturers and individual countries or through the COVAX Facility. This is another lesson learned, highlighting the importance of developing clear disclosure policies in advance to promote transparency for the public and predictability for manufacturers. For example, one key informant from an international organization stated:

*“There is a need for a clear disclosure policy where we agree this will be disclosed and comply to it we agree on type of information that will be disclosed and that is enforced.”*

This was echoed in a report by Transparency International, which advocated for the publication of vaccine contracts (with justifications provided for any redactions) to promote vaccine pricing transparency [16]. Donor agencies and international organizations were identified as key stakeholders to push for increased transparency during the contract negotiation process [33]. A key informant from an NGO shared:

*“The World Bank has provisions for ensuring information is made public. While the World Bank doesn’t itself have this mechanism*,* countries that receive support from the Bank must make information publicly available.…some of the greatest pressure for integrity*,* accountability and transparency can come from international funding organizations. If government is obstructive and citizens are weak or if citizens don’t care how the government is spending the money*,* then it’s an issue and in this case*,* an international organization can apply pressure.… International organizations and international financial institutions have a unique capacity to influence anticorruption in vaccine distribution.”*

Compliance with existing good practices frameworks in the public procurement space was also recommended, including the *G20 COVID-19 Good Practices Compendium on Combating Corruption in the Response to COVID-19* emergency procurement chapters [57]. Digital solutions, such as open contracting and e-procurement, were proposed as additional transparency mechanisms [12, 25] and are discussed in more detail below.

### Pooled procurement

Pooled procurement for vaccine purchases has emerged as a vital strategy for improving pandemic preparedness, especially for low- and middle-income countries (LMICs) facing resource limitations. Pooled procurement involves buyers collaborating to consolidate their purchases, often to leverage demand aggregation to drive price reductions [58]. This procurement mechanism enables LMICs to navigate the complexities of procurement by centralizing risk management, leveraging international partnerships, and benefiting from shared governance frameworks provided by facilitating organizations [59]. Several key informants highlighted the benefits of pooled procurement, with one international organization consultant explaining that before COVID-19:

Pooled procurement [run by] UNICEF and PAHO helps ensure that the quality of pharmaceuticals is good, which is useful because many LMICs don’t have mechanisms to do quality checks.

To illustrate, the Strategic Fund for Essential Health Medicines and Supplies, initiated in 2000 by PAHO, is a pooled procurement mechanism for vital medicines and supplies across the Americas [60]. Through leveraging diverse technical expertise, fostering multilateral cooperation, and nurturing established partnerships, the Fund aims to enhance access to high-quality, safe, and cost-effective health products. During the COVID-19 pandemic, PAHO and its member states used the Strategic Fund to address health emergencies, sustain critical public health programs, prevent shortages, manage supply chain disruptions, and procure essential supplies worth US$300 million [60]. This reportedly helped 40 million individuals by providing diagnostic tests, personal protective equipment (PPE), and intensive care medicines [61].

### Direct civil society engagement

Effective pandemic preparedness requires the active engagement of civil society to ensure transparency, accountability, and public trust in health and pharmaceutical systems. Several key informants attributed the high risk of corruption in the deployment of COVID-19 vaccines to the exclusion of civil society from the procurement and distribution process, with one key informant from an NGO commenting that:

[it is] highly valuable and important to have citizen engagement at the grassroots level to promote accountability and transparency. It is vital to understand what is happening at the grassroots level.

Civil society organizations were identified as being able to play a key role in supporting government monitoring and reporting efforts [12]. For example, the International Federation of Red Cross and Red Crescent Societies partnered with the World Bank to act as a third-party monitoring agency in Lebanon to ensure compliance with COVID-19 deployment plans [61]. Civil society can also play a role by engaging with pharmaceutical companies on transparency initiatives and applying social pressure to ensure they fulfill their contractual responsibilities [32, 33]. An example of how civil society organizations can advance transparency was shared by a key informant from an NGO:

In Argentina, the civil society observatory takes government information (e.g., the total number of contracts for medical supplies and equipment) and makes it publicly available. This helps the public get information that is readily available. The Observatory became the most important information source during COVID-19.

For civil society engagement to be successful, reporting channels must be readily available to facilitate effective and timely reporting of corruption by the public [12]. Additionally, public information campaigns run by civil society organizations can educate communities on the risks of corruption in the vaccine distribution process and promote greater vigilance and accountability [31].

### Digital solutions to promote transparency

Digital solutions have become indispensable tools in promoting transparency and improving pandemic preparedness. Technologies such as track-and-trace systems (e.g., barcodes, QR codes) are effective in monitoring supplies in transit, reducing the risks of theft, diversion, and entry of substandard or falsified vaccines into supply chains [12, 25, 62]. For instance, using QR codes to verify vaccine certificates digitally was widely adopted during the COVID-19 pandemic. A key informant, who consulted for an international organization, stated:

All countries introduced QR codes for the vaccine certificates so people realized if they don’t have the certificate generated by the system, they could not get away with fake certificates.

Another key informant working at an international organization country office in India described how CoWIN (Covid Vaccine Intelligence Network), the Indian Government’s web portal for COVID-19 vaccination registration, played a crucial role in tracking doses from procurement to patient delivery:

CoWIN allowed for increased ownership down to beneficiary; it allowed government to allow beneficiaries to register themselves. The web portal allowed citizens to track doses. Being able to digitize these things and tag each beneficiary to each dose supported increasing the sensitivity of the surveillance system. It also tracked and sent reminders to beneficiaries once their second dose was due. The system was done in a transparent way.

While ultimately unsuccessful, attempts in Nigeria to develop an online procurement portal also demonstrate how digital solutions can be leveraged to increase transparency and public monitoring of government procurement activities. One key informant representing Nigeria commented:

There was a platform called National Nigerian Open Contracting Portal (NOCOPO) which was supposed to publish all COVID-19 procurement processes, but that platform was not fully functional. I got discouraged because it didn’t function, I can’t get any data. It was established that all procurement processes had to be published, but this was not the case.

Blockchain-based solutions were also proposed to improve transparency in the COVID-19 vaccine supply chain [31, 63–67]. Given that public blockchains comprise a decentralized ledger, multiple sources suggested using these to enable governments to monitor whether shipments were delivered to the right place at the right time and help vaccine recipients verify whether their received doses are legitimate [64]. Blockchain’s ability to eliminate human interference by automating the vaccine management and delivery processes was highlighted as one of its main benefits [62, 63].

At the global level, digital dashboards have emerged as critical resources for pandemic preparedness, with several international organizations implementing online public dashboards to increase access to COVID-19 vaccine related data. For example, as part of the SDG 3 Global Action Plan for Healthy Lives and Well-being, the UNDP in partnership with the WHO and the University of Oxford developed the Global Dashboard for Vaccine Equity that provided data on the global COVID-19 vaccine rollout combined with up-to-date socio-economic information (e.g., economic support index, current health expenditure per capita) [48]. This dashboard served as a tool to highlight the importance of advancing vaccine equity and understand ways to help address it. The COVID-19 Market Dashboard managed by UNICEF is another example of a digital platform that can enhance transparency, as it provides publicly accessible information on how allocation was operationalized, manufacturing agreements, and reported vaccine prices [41]. Importantly, information on vaccine prices and manufacturing agreements can also be leveraged during pandemic preparedness efforts to promote transparency and accountability. Public access to such data enables citizens and advocacy groups to monitor the equitable distribution of resources, ensuring that international organizations and governments prioritize fairness and efficiency in their responses to health crises [16].

One key informant, who was an international organization consultant, also underscored the importance of not over-relying on digital transparency mechanisms, particularly in terms of equity:

Some countries put very rigid online registration systems; this created a digital divide. It benefitted people who have access to internet and smart devices, but older people and rural areas got left out in this registration process, so this created inequities.

Key informants highlighted that digital solutions must be integrated into pandemic preparedness plans in ways that prioritize equity. Ensuring access to these tools in under-resourced and rural areas, while incorporating non-digital alternatives will be essential to achieving inclusive and transparent health responses.

## Discussion

Our descriptive, qualitative study identified corruption risks in the procurement and distribution of COVID-19 vaccines and the lessons learned that highlight what ACTA mechanisms can reduce these risks. Consistent with prior literature on the procurement of health products [18, 25, 68], our analysis of the literature and key informant interviews revealed that, while vaccine delivery is susceptible to corruption, the procurement process of COVID-19 vaccines holds an even greater risk of corruption. Our study further illustrated that procurement corruption risks were significantly heightened during COVID-19 due to factors unique to the pandemic context, such as the urgent need for governments to rapidly acquire vaccines in high demand but limited supply. Several key informants also noted that compared to other healthcare products, such as medicines for HIV, there was substantially less transparency in the procurement of COVID-19 vaccines. The opacity of the procurement process, including vaccine prices and vaccine contracts between COVAX and donor/recipient countries, was inconsistent across international and national levels, likely driven by the need for a swift response to the public health emergency. Although most key informants reported the increased prevalence of corruption during COVID-19, they acknowledged that the unprecedented challenges the pandemic posed to health and economic systems made it extremely difficult to mitigate corruption risks specific to the pharmaceutical system.

Our study highlighted several ways to minimize corruption risks in the procurement and distribution of vaccines amidst public health crises. Among the ACTA mechanisms for vaccine deployment, transparency was the most endorsed in both the literature and by key informants. This likely explains the predominance of transparency mechanisms implemented during the deployment of COVID-19 vaccines, such as India’s CoWIN platform and UNICEF’s COVID-19 Market Dashboard [41]. A few key informants, however, emphasized that transparency alone is insufficient without accompanying accountability measures to enforce sanctions for corruption. These findings support existing literature on the need for stronger transparency and accountability structures in the procurement and distribution of COVID-19 vaccines specifically [14] and in the pharmaceutical system broadly [69]. Notably, our study found that corruption in COVID-19 vaccine deployment was opportunistic, occurring sporadically at micro- or meso- levels, rather than being a systems-level issue.

Several important lessons emerged from our study. First, as highlighted in our findings, involving civil society organizations is critical, as they can support government monitoring and reporting efforts at the local level, thereby strengthening governance and improving public service delivery. This aligns with previous studies that suggest social accountability or community-based monitoring can reduce corruption risks [70]. Although not identified in our study, the implementation of whistleblowing mechanisms is another effective method to reduce the risks of individual acts of corruption. Whistleblowing mechanisms (and whistleblower protection) can help identify alleged incidents of corruption in the procurement process, especially considering that current policies and procedures many organizations have put in place have not been sufficient to lower the risk of corruption [71, 74] and should be incorporated into solutions that aim to improve the pharmaceutical supply chain and procurement process. Whistleblowing exposes incidents of corruption with the goal that public exposure will discourage corrupt behavior [72]. Whistleblower mechanisms alone are not sufficient and should be accompanied by whistleblower protection programs or legislation (e.g., UN’s Protection Against Retaliation Policy). A key deterrent to reporting wrongdoing is the lack of legal protection from retaliation. Prioritizing whistleblower protection not only shields the whistleblower from negative consequences but also encourages others to speak out against corruption [72–74]. Therefore, pandemic preparedness should emphasize the development of emergency reporting channels for corruption to enhance accessibility and effectiveness.

Interestingly, our findings show that lessons learned from previous epidemics and pandemics were not adequately integrated into the COVID-19 response. Lessons learned then were forgotten. In line with our finding during COVID-19, past epidemics like Ebola and H1N1 illuminated the critical importance of building public trust in government as a key factor in controlling the spread of disease. For example, during the Ebola outbreak in Liberia and the Democratic Republic of the Congo, public trust in government institutions significantly increased compliance with public health regulations to mitigate the spread of the virus [50]. Similarly, during the 2009 H1N1 influenza epidemic, public trust in federal authorities in Italy, the Netherlands, and Switzerland contributed to improved sanitation behaviors, such as hand washing, social distancing, and acceptance of the H1N1 vaccine [50]. Undoubtedly, high levels of public trust are critical for an effective response to public health emergencies. Therefore, future emergency preparedness plans should include strategies to enhance public trust at both global and national levels.

Furthermore, the Global Fund to Fight AIDS, Tuberculosis and Malaria’s (The Global Fund) response to global epidemic of HIV/AIDS has shown the importance of engaging the civil society at all levels of the response. More specifically, the Global Fund was established in 2002 in response to civil society organizations and communities calling for more resources to tackle HIV. The Fund has since involved communities and civil society in governance at the international level, allocating three voting seats on its Board to community and civil society representatives, with two seats held by NGOs (one from a developing country and one from developed country) and one seat held by communities affected by AIDS, tuberculosis, and malaria [73]. At the country-level, the Fund involves civil society through their membership in the Country Coordinating Mechanism, where they contribute to funding requests and oversee the implementation of funds [75]. Future global initiatives aimed at combating pandemics, such as COVAX, should follow a similar civil society engagement model to that of the Global Fund.

### Limitations

This study has a few limitations. Our intention was to interview representatives from COVAX to gain insights specific to this new global initiative, as well as members from both COVAX donor and recipient countries to better capture corruption risks across different health, economic and political systems. Nonetheless, despite the research team’s best efforts, the response rates from representatives directly involved with COVAX and members of donor countries were extremely low, which created an overrepresentation of international organizations that were not directly involved with COVAX and recipient countries in our sample. Of the individuals who responded but declined the interview request, most could either not dedicate time or were not able to discuss information about their organization. Specifically, individuals who had worked directly with COVAX reported they had signed a non-disclosure agreement that does not permit them to share information about the initiative. Consequently, there are limited insights from the COVAX Facility itself. Another limitation of this study is the potential for selection and response bias. Those who chose to participate may have different perspectives than those who declined or were unable to respond, and the self-selecting nature of the sample means that the views captured may not fully represent the broader range of stakeholders involved in COVAX. Additionally, there is the potential for bias in the responses from participants. Some individuals may have had a vested interest in presenting COVAX or its processes in a favorable light, while others may have approached the topic with concerns or criticisms based on prior experiences, which could have influenced their responses. To mitigate these biases, interview data were triangulated with other relevant literature to ensure a more balanced and comprehensive analysis. In time, we may be able to get better access to information about lessons learned and forgotten during COVID-19. To achieve this, future work on COVAX should explore donor countries’ views and perceptions to understand the corruption risks in vaccine procurement and deployment in the context of a pandemic in high-income countries.

## Conclusion

Our study contributes to the growing body of literature on lessons learned from the COVID-19 pandemic, one of the most significant public health crises in history. Our findings highlight notable shortcomings in the implementation of ACTA mechanisms within the COVID-19 vaccine deployment process. These shortcomings not only highlight vulnerabilities in procurement and distribution systems but also emphasize the urgent need to strengthen transparency, accountability, and civil society involvement in health system governance. The COVID-19 experience offers valuable insights that are essential for building more robust frameworks to address corruption risks in future public health emergencies. As we move forward, it is imperative that we integrate these lessons into global health policies and pandemic preparedness strategies to ensure greater resilience and equity in future responses. The implementation of ACTA mechanisms will be pivotal in safeguarding the integrity of health systems, reducing corruption, and ensuring that health interventions reach those most in need in times of crisis.

## Electronic supplementary material

Below is the link to the electronic supplementary material.

Supplementary Material 1

## Data Availability

Data for the literature review in this manuscript were collected through publicly available websites. Specific documents cited in our literature review are referenced with URLs in the reference section of the manuscript. However, data for the key informant interviews are not available as per the University of Toronto ethics approval, which states that key informant interviews are strictly confidential.
