# Supplementary material for: Corruption risks in COVID-19 vaccine deployment: lessons learned for future pandemic preparedness
Source: Global Health. 2025 Mar 7;21:8. doi: 10.1186/s12992-025-01096-6 (PMC11887148; doi:10.1186/s12992-025-01096-6)
Supplement: Supplementary file 1 — Supplementary Material 1 [file 12992_2025_1096_MOESM1_ESM.docx]

**Appendix A**

Interview Guide

1. Please describe your responsibilities at your organization.
    i. Please describe the work that you do and how you are engaged with the
    COVID-19 vaccine deployment process, including procurement and/or
    distribution.
2. **If it is a country representative:** Please describe the process or purchasing arrangements of how your population is getting access to the COVID-19 vaccines.
   - 1. Is your country receiving vaccines from the COVAX facility?
     2. What % of covid vaccines are purchased directly from manufacturers and what % are obtained from COVAX?
     3. Is your country receiving donated vaccines or through other pooled mechanisms?
     4. Who are some of the stakeholders involved in this process?

***Move on to question 3 if it is a member of the COVAX Facility.***

1. **If it is a member of the COVAX facility**: Please tell me about some of the ways that the COVAX Facility is ensuring equitable access to the COVID-19 vaccines globally?
   - 1. Who are some of the stakeholders involved during this process?

***Skip question 3 for country representatives.***

1. What are the anti-corruption, transparency, and accountability mechanisms
   in place for your organization’s or country’s approach to getting access to COVID-19 vaccines?
2. What are the strengths and weaknesses of this approach in terms of ACTA mechanisms?
3. How has the COVID-19 pandemic affected the supply chain of COVID-19 vaccines in your country or organization?
   - 1. Has there been mismanagement in the deployment of the vaccines?
     2. Are you aware of any cases of diversion of vaccines or falsified vaccines infiltrating supply chains or embezzlement of finances?
     3. (If there has been) can you give me an example?
     4. What are some ways the COVAX facility, or your country are dealing with mismanagement issues?
4. Have you observed any corruption risks within the vaccine procurement or distribution processes at your organization, in your country, or more generally?
5. Please describe facilitators and barriers that either help or hinder accountability and transparency in the procurement and distribution of COVID-19 vaccines at the COVAX facility or your country?
   - 1. Can you speak to if/how these facilitators and barriers differ from routine times?
     2. Can you speak to if/how these facilitators and barriers have changed throughout the pandemic?
6. How does a lack of transparency and accountability in procurement of COVID-19 vaccines impact equity in access to these vaccines at the global level and/or national level?
   - 1. Who is most adversely affected by corruption of supply chains?
7. Moving forward, what could be done to improve accountability and transparency in the procurement and/or distribution of the COVID-19 vaccines at your organization or in your country specifically and in the pharmaceutical sector more generally?
8. Are there specific documents about COVID-19 vaccine procurement and distribution in your organization or country that you think we should look at for our research?
9. Is there any information that you feel is relevant that you would like to add to this discussion?
10. Can you please provide us with 2 names of individuals, either someone who works at the same organization as you or someone who could provide us with more information on the topic of COVID-19 vaccine procurement and distribution?
